# Acute leukemia in pregnancy: a single institutional experience with 21 cases at 10 years and a review of the literature

**DOI:** 10.1080/07853890.2021.1908586

**Published:** 2021-04-06

**Authors:** Dengqin Zhu, Doudou Tang, Xiaoshan Chai, Guangsen Zhang, Yewei Wang

**Affiliations:** a Department of Hematology, The Second Xiangya Hospital, Central South University, Changsha, China; b Department of Respiratory and Critical Care Medicine, the Second Xiangya Hospital, Hunan Centre for Evidence-based Medicine, Central South University, Changsha, China; c Department of Obstetrics and Gynecology, the Second Xiangya Hospital, Central South University, Changsha, China; d Institute of Molecular Hematology, Central South University, Changsha, China

**Keywords:** Acute leukaemia, pregnancy, chemotherapy, acute myeloid leukaemia, acute lymphoblastic leukaemia

## Abstract

**Introduction:**

Acute leukemia (AL) occurring in pregnancy is extremely rare, and its treatment is a clinical dilemma.

**Methods:**

We retrospectively reviewed the medical records of our hospital from 2010 to 2019.

**Results:**

Twenty-one patients were diagnosed with AL during pregnancy. Of whom, eighteen had acute myeloid leukemia, and 3 had acute lymphoblastic leukemia. Six, eight and seven patients were diagnosed during the first, second, and third trimester, respectively. Six of the 21 patients experienced therapeutic abortion and 1 had spontaneous abortion, whereas 9 gave birth to healthy babies (4 through vaginal deliveries and 5 with Caesarean sections). Four babies had been exposed to chemotherapeutic agents, but no congenital malformations were observed. Sixteen patients received chemotherapy, while 4 patients died before chemotherapy and one was discharged after refusing chemotherapy. The complete remission rate of the 10 patients who began chemotherapy immediately after diagnosis was 80%, compared with 66.7% in the 6 patients who started chemotherapy after abortion or delivery. Three remain alive.

**Conclusions:**

In general, initiation of chemotherapy as early as possible may increase the CR rate. Combined with literature data, we proposed that, for patients diagnosed in early and late stages of pregnancy (>30 weeks), elective termination or induced delivery before chemotherapy may be a good choice for better maternal (and fetal) outcome.

**KEY MESSAGES**
- Acute leukaemia diagnosed in pregnancy is extremely rare, and its treatment is a clinical dilemma.
- In general, initiation of chemotherapy as early as possible may increase the CR rate.
- For patients who are diagnosed in the first trimester or late stage of pregnancy (>30 weeks), elective termination or induced delivery before starting chemotherapy may be a good choice for better maternal (and fetal) outcome.

## Introduction

Acute leukemia (AL) is a malignant blood disease that arises from either the myeloid cells (acute myeloid leukemia, AML) or the lymphoid cells (acute lymphoblastic leukemia, ALL). The incidence of AL increases dramatically with age and peaks at 80–85 [1]. Although elderly patients are at high risk of AL, the disease can also be observed in women of childbearing age. The incidence of leukemia in pregnancy has been reported as one in 75,000–100,000 [2–4]. During pregnancy, some of the early features of AL, such as fatigue and shortness of breath, or alterations of peripheral blood counts, as anemia and thrombocytopenia, may be interpreted as pregnancy-related symptoms, leading to delayed diagnosis and inappropriate therapy [5,6]. If it is untreated immediately, the disease would result in rapid maternal and fetal mortality [7,8]. In addition, delaying induction chemotherapy negatively impacts the likelihood of remission [9]. Although a guideline for the diagnosis and management of acute myeloid leukemia in pregnancy had been published by British Committee for Standards in Hematology in 2015 [7], management decision is influenced by many factors, such as the type of leukemia, severity of illness, financial situation, and patient’s personal beliefs.

In this study, we reported 21 cases of acute leukemia diagnosed during pregnancy between April 2010 and June 2019, and analyzed their clinical characteristics, treatments and outcomes, in order to make useful suggestions for the management of acute leukemia in pregnancy.

### Patients and methods

After approval by the Institutional Review Board, information was collected retrospectively using institutional electronic medical records on all patients with AL who met the diagnosis during pregnancy in the Second Xiangya Hospital of Central South University from April 2010 to June 2019. All procedures followed were in accordance with the ethical standards of the Helsinki Declaration of 1975, as revised in 2013.

The diagnosis and classification of AL were based on the French-American-British criteria and the revised WHO criteria, 2016 [10,11]. According to the WHO criteria, diagnosis of AL requires at least 20% blasts among no erythroid cells in bone marrow or blood. Treatment of patients with AL is divided into two chemotherapy phases: remission induction and consolidation. After two cycles of induction chemotherapy, most patients achieved a status with no symptoms or signs of the disease, fewer than 5% blast cells in bone marrow and normal blood cell counts, which is named complete remission (CR). Patients who do not achieve complete remission after induction chemotherapy are usually diagnosed as having “refractory AL”.

Inclusion and exclusion criteria: only patients with acute leukemia diagnosed during pregnancy were included; Patients with pregnancies that occurred during or after treatment for AL were excluded.

The information we collected includes the patient’s age at diagnosis, gestational age at diagnosis, peripheral blood counts at diagnosis, blasts in bone marrow, cytogenetics and molecular markers, whether or not delivery, type of delivery or abortion, postnatal condition, induction therapy regimen, response to chemotherapy, maternal and fetal outcomes. The first trimester is defined as before the end of week 12 and the third trimester is after week 27 of pregnancy.

## Results

### Characteristics of patients

We identified 21 patients with acute leukemia occurring during pregnancy. Eighteen patients had AML, and 3 had ALL. The median age at leukemia diagnosis was 28 years (range 19–41 years). The median gestational age at diagnosis was 22 weeks (range <4 to 36 weeks). The details of the patients were shown in Table 1 and Supplementary Table S1. As described in Table 2, six, eight and seven patients were diagnosed during the first, second, and third trimester, respectively. Among the 21 patients, six patients experienced elective abortion (5 cases of medical abortion and 1 of curettage abortion) and 1 had spontaneous abortion, 9 gave birth to babies (4 through vaginal deliveries and 5 underwent Caesarean sections), and 5 patients died (four died before induction chemotherapy, one declined chemotherapy and died after discharge). The interval between diagnosis of AL and the commencement of chemotherapy ranged from 1 day to 11 days (median, 5 days). The mean length of follow-up was 23.6 months. Five patients had severe extra-hematologic complications (WHO Grade ≥ 3) after induction chemotherapy, including infections (4 patients) and hemorrhage (1 patient, No.9). No venous or arterial thrombus was found.

**Table 1.** Clinical features of 21 patients with acute leukemia diagnosed during pregnancy.

Patient No.	Age at diagnosis (years)	Pregnancy No.	Gestation at diagnosis (weeks)	AL type	Induction therapy	Pregnancy outcome	Fetal outcome	Response to therapy	Chemotherapy first	Delivery or abortion first	Interval between diagnosis and chemotherapy (days)	Survival time after diagnosis (months)	Patient outcome
1	33	G5P1	26w + 4d	AML-M5	DNR + AraC	Caesarean section	Premature birth	CR	Yes	No	4	23	Dead: pulmonary infection
2	25	G2P1	＜4w	AML-M3	DNR + ATRA	Therapeutic abortion	–	CR	Yes	No	2	109	Alive
3	30	G2P1	29w + 6d	AML-M5	DNR + AraC	Caesarean section	Premature birth	Refractory	No	Yes	11	3	Dead: resistance
4	19	G1P0	＜4w	ALL	–	Hospital discharge	–	Death	No			1	Dead: declined therapy
5	33	G3P1	34w + 1d	ALL	DNR + VCR + CPM + Pred	Caesarean section	Term infant	CR	Yes	No	5	8	Dead: pulmonary infection
6	34	G7P1	11w + 5d	AML-M2	DNR + AraC	Spontaneous abortion	–	Refractory	Yes	No	4	4	Dead: resistance
7	28	G1P0	14w + 1d	AML-M4	DNR + AraC	Therapeutic abortion	–	CR	Yes	No	4	26	Dead: infection
8	39	G2P1	16w	AML-M4	DNR + AraC	Therapeutic abortion	–	CR	Yes	No	5	77	Alive
9	26	G2P0	16w	AML-M1	IDA + AraC	Therapeutic abortion	–	CR	Yes	No	5	7	Dead: haemorrhage
10	23	G1P0	4w	AML-M4	–	Death	–	Death	Yes	No	4	0	Dead: intracranial haemorrhage
11	40	G3P2	6w + 3d	AML-M4	DNR + AraC	Therapeutic abortion	–	Refractory	No	Yes	11	2.5	Dead: resistance
12	24	G4P1	8w	AML-M4	–	Death	–	Death	No			0	Dead: intracranial haemorrhage
13	28	G3P0	22w	AML-M5	–	Death	–	Death	No			0	Dead: massive haemoptysis
14	27	G1P0	34w	AML-M3	ATRA + DNR	Vaginal delivery	Term infant	CR	Yes	No	6	47	Dead: refractory leukaemia
15	41	G6P1	27w + 4d	ALL	DNR + VCR + CPM + Pred	Vaginal delivery	Premature birth	CR	Yes	No	4	16	Dead: refractory leukaemia
16	24	G2P1	35w	AML-M3	ARTA + IDA	Vaginal delivery	Term infant	CR	No	Yes	1	7	Dead: pulmonary infection
17	26	G4P1	36w + 6d	AML-M2	DNR + AraC	Caesarean section	Term infant	CR	No	Yes	10	50	Alive
18	30	G2P1	22w	AML-M3	ATRA	Death	–	Death	No		1	0	Dead: intracranial haemorrhage
19	29	G2P1	36w	AML-M2	DNR + AraC	Caesarean section	Term infant	Refractory	No	Yes	9	2	Dead: resistance
20	27	G1P0	30w	AML-M2	IDA + AraC	Vaginal delivery	Premature birth	CR	No	Yes	5	7	Dead: aGVHD after HSCT
21	26	G2P1	18w + 3d	AML-M4	DNR + AraC	Therapeutic abortion	-	CR	Yes	No	10	11	Dead: infection

AL: Acute leukemia; AML: Acute myeloid leukemia; ALL: Acute lymphoblastic leukemia; DNR: Daunorubicin; AraC: Cytarabine; ATRA: All-trans retinoic acid; VCR: Vincristine; CPM: Cyclophosphamide; Pred: Prednisone; IDA: Idarubicin; aGVHD: Acute graft-versus-host disease; HSCT: Hematopoietic stem cell transplantation.

**Table 2.** Characteristics of different stages of pregnancy.

Characteristics	Trimester in which AL was diagnosed		
	First trimester	Second trimester	Third trimester
Case number	6	8	7
Median age (years)	24.5	29	27
Type of leukemia			
AML	5	7	6
ALL	1	1	1
Previous pregnancy			
Yes	4	7	5
No	2	1	2
Termination of pregnancy			
Therapeutic abortion	2	4	0
Spontaneous abortion	1	0	0
Caesarean sections	0	1	4
Vaginal deliveries	0	1	3
Hospital discharge	1	0	0
Death	2	2	0
Response to induction chemotherapy			
CR	1	6	5
Refractory	2	0	2
Recurrence			
Yes	0	5	3
No	1	1	2

AL: Acute leukaemia; AML: Acute myeloid leukemia; ALL: Acute lymphoblastic leukemia.

### Acute promyelocytic leukemia

Four patients were diagnosed with acute promyelocytic leukaemia (APL), the M3 subtype of AML. Three of them received all-trans retinoic acid (ATRA) and daunorubicin (DNR) or ATRA and idarubicin (IDA) therapy, and achieved CR. Patient No.2, diagnosed in the first trimester, received ATRA and DNR therapy followed by therapeutic abortion. She achieved CR and completed consolidation and maintenance chemotherapy. This patient is still alive. Patient No.18, diagnosed in the second trimester, died due to intracranial hemorrhage before induction therapy. Two patients were diagnosed during the third trimester. One (patient No.14) was diagnosed with high-risk APL (WBC count >10 × 10^9^/L) and started induction therapy (ATRA + DNR) first. She experienced an induced vaginal delivery at 38 weeks when neutrophil and platelet recovery. The second patient (patient No.16) opted to receive single-agent ATRA and underwent an induced vaginal delivery of a full-term baby, then proceeded to initiation of IDA chemotherapy because of a marked increase in WBC numbers. Both of them obtained a CR. Unfortunately, patient No.14 relapsed three times and died of refractory leukemia at 47 months after diagnosis. Patient No.16 relapsed at 10 months after diagnosis, and died from pulmonary infection after re-induction chemotherapy. Both patient No.14 and No.16 gave birth to healthy babies.

### Acute myeloid leukemia

A total of 14 patients were diagnosed with non-APL AML. Eleven patients received induction chemotherapy with DNR and cytarabine (Ara-C) (9 patients) or IDA and cytarabine regimen (2 patients). Seven of the 11 cases achieved CR, but 4 patients eventually relapsed and died. Two patients remain alive. The remaining 4 cases who received chemotherapy had refractory disease.

### Trimester-specific outcomes

#### First trimester

Four of the 14 AML patients were diagnosed in the first trimester of pregnancy. Two patients received induction chemotherapy (DNR + Ara-C), one of them (patient No.6) began therapy immediately after diagnosis and had a spontaneous abortion, the other one (patient No.11) experienced elective termination first and then initiated chemotherapy. Unfortunately, both of them were refractory to induction chemotherapy, and died 4 months and 2.5 months after diagnosis respectively. Two patients (patient No.10 and No.12) died from intracranial hemorrhage before chemotherapy. None of the patients diagnosed in the first trimester made it to a successful delivery.

#### Second trimester

Six patients were diagnosed with AML during their second trimester. Five AML patients received chemotherapy (4 with DNR + Ara-C and 1 with IDA + Ara-C), and all of them obtained CR. Unfortunately, 4 patients developed recurrent disease and died. Four patients diagnosed before 20 weeks of gestation opted to initiate chemotherapy after diagnosis and underwent elective abortion after neutrophil and platelet recovery. One patient (patient No.1) diagnosed at 26 weeks began induction therapy first and delivered a low-birth-weight baby at 30 weeks by Caesarean section. Patient No.13 died from massive haemoptysis before induction chemotherapy.

#### Third trimester

Four patients were diagnosed in the third trimester. All of them received induction chemotherapy after delivery (3 with DNR + Ara-C and 1 with IDA + Ara-C). Three of them underwent Caesarean section and 1 had an induced vaginal delivery. They gave birth to 2 preterm (patient No.3 and No.20) and 2 full-term infants (patient No.17 and No.19). Two patients (patient No.17 and No.20) achieved CR, one of them (patient No.20) died after allogeneic hematopoietic stem cell transplantation due to acute gut graft-versus-host disease at 7 months after diagnosis. The other two patients (patient No.3 and No.19) died of refractory AML. All of the 4 patients gave birth to healthy babies, and no congenital malformations were observed.

#### Acute lymphoblastic leukemia

Three patients were diagnosed with Philadelphia chromosome negative B cell ALL, without central nervous system involvement. One patient (patient No.4) diagnosed in the first trimester of pregnancy, declined therapy and died shortly after discharge. The other 2 patients (patient No.5 and No.15) diagnosed at 34 weeks and 27 weeks respectively, opted for immediate induction chemotherapy (Cyclophosphamide + Daunorubicin + Vincristine + Prednisone), then underwent induced vaginal delivery and Caesarean section respectively after neutrophil and platelet recovery. Both of them achieved CR, but relapsed. Patient No.15 died of refractory ALL at 16 months and patient No.5 died of respiratory failure caused by pulmonary infection at 8 months. Two babies were healthy and no malformation.

#### Neonatal outcomes

All of the 9 babies were alive (Summarized in Table 3). Four were premature and 5 were full-term infants. Four of the 9 newborns had been exposed to chemotherapeutic agents (2 diagnosed in the second trimester and 2 diagnosed during the third trimester). The 1-minute Apgar scores for the 9 newborns were greater than or equal to 7, and the 5-minute Apgar scores were greater than or equal to 8. No foetal malformations were found in our study. The median length of follow-up was 42 months. Long-term follow-up showed that there were no abnormal blood images, no abnormal cardiac function and other discomforts, and normal growth and development.

**Table 3.** Characteristics of 9 newborns.

Patient No.	Delivery mode	Delivery time	Gender	Weight (g)	Apgar score		Receive chemotherapy before delivery	Follow up duration (months)
					One minutes after birth	Five minutes after birth		
1	Caesarean sections	Premature birth	Female	2000	8	9	Yes	108
3	Caesarean sections	Premature birth	Male	1850	7	8	No	98
5	Caesarean sections	Term infant	Male	2900	9	10	Yes	84
14	Vaginal deliveries	Term infant	Male	2935	10	10	Yes	44
15	Vaginal deliveries	Premature birth	Female	2000	8	9	Yes	42
16	Vaginal deliveries	Term infant	Female	2600	9	9	No	42
17	Caesarean sections	Term infant	Male	3290	8	10	No	36
19	Caesarean sections	Term infant	Female	2730	8	10	No	30
20	Vaginal deliveries	Premature birth	Male	1760	8	8	No	15

## Discussion

Since the first report of pregnancy with acute leukemia was published in the year 1845, patients with gravid acute leukemia had been increasingly reported [12]. However, due to the rarity of pregnancy complicated with acute leukemia, relevant literature had been published mainly in the form of small series and case reports. We summarized literature (cases ≥ 5) regarding acute leukemia diagnosed in pregnancy (Table 4) [2,13–24]. In this study, we reviewed retrospectively and analyzed 21 cases of acute leukemia during pregnancy. Our results were in accordance with previous results that AML accounts for more than two-thirds of acute leukemia during pregnancy [14,25]. Patients diagnosed in the first, second and third trimester were 6, 8 and 7, which were also similar to previous report [22]. Our result of CR rate was at the same level as previously reported results [17,22], and delayed induction chemotherapy reduced the CR rate.

**Table 4.** Literature (Cases ≥ 5) regarding acute leukemia diagnosed in pregnancy.

References	AL type & No. of cases	Patient’s outcome	Pregnancy and fetal outcome
Catanzarite and Ferguson [13]	AL, 47	40 treated (survival > 6 months); 7 untreated (1 survival)	5 TA, 3 perinatal demises, 1 live-born infant, 31 LI; 1 TA, 2 perinatal demises, 4 LI
Reynoso et al. [14]	AL, 58	53 treated; No mortality	50 LI (28 premature); 1 CM
Zuazu et al. [15]	AML, 8; ALL, 1	2 deaths	2 SA; 1 TA;1 C-section; 3 VD
Fadilah et al. [16]	APL 16	No mortality	16 LI (2 cardiac symptoms)
Ali et al. [2]	AML 6; ALL 2	3 CR; 5 died	2 SA; 5 TA;1 ID
Chelghoum et al. [17]	AML 31; ALL 6	34 CR	13 VD (14 LI); 9 C-section; 2 SA; 13 TA
Aviles et al. [18]	AML 8; ALL 6	10 CR (71%)	13 LI; 1 SA
Saleh et al. [19]	AML 15; ALL 6	14 died	10 LI; 3 SA; 8 TA
Nakajima et al. [20]	AML 8; ALL 3	10 CR; 8 alive; 3 died	4 TA; 1 SA; 1 ID; 5 LI
Sanz et al. [21]	APL 14	92% CR; 11 alive; 3 died	4 TA;1 SA; 1 ID; 8 LI
Farhadfar et al. [22]	AML 18; ALL 5	78% CR; 7 alive; 15 died	5 TA; 6 SA; 12 LI
Fracchiolla et al. [23]	AML 5	4 alive	1 TA; 1 ID; 3 LI
Mabed et al. [24]	AML 18; ALL 9	15 CR; 14 died.	3 SA; 3 TA; 2 ID; 19 LI

AL: acute leukemia; AML: acute myeloid leukemia; ALL: acute lymphoblastic leukemia; APL: acute promyelocytic leukemia; CR: complete remission.

TA: therapeutic abortion; LI: live infant; CM: congenital malformation; SA: spontaneous abortion; VD: vaginal delivery; ID: Intrauterine death; C-section: caesarean section.

Although in general, delayed initiation of chemotherapy is associated with poor maternal outcome [9], a slight delay in treatment to allow for delivery first may be reasonable for patients diagnosed in the late stage of pregnancy (>30 weeks) [17]. There are many reasons support this suggestion. First, though no major malformations were observed in infants who were exposed to chemotherapy in the third trimester, cases of hematopoietic suppression, growth restriction, intellectual impairment and reduced fertility have been reported [14,26–31]. In addition, when cytotoxic chemotherapeutic agents are administered after 30 weeks, the delivery may be initiated during the bone marrow suppression period that may increase the incidence of infection and hemorrhage [32]. Furthermore, there has been reported that neonatal survival rates are higher than 90% when delivery at or after 28 weeks’ gestation in most comprehensive hospitals, and even higher (>95%) when delivery at or beyond 32 weeks’ gestation [7]. As a result, delivery before initiation of chemotherapy when the diagnosis is made after 30–32 weeks’ gestation, has been proposed in the 2015 guideline for AML in pregnancy [7]. When patients are diagnosed with AL after 30 weeks’ gestation, delivery first will minimize fetus chemotherapy exposure while keeping a high neonatal survival rate. In our series, patients diagnosed in advanced gestational age (>30 weeks) after 2015, had induced vaginal delivery or Caesarean section first, and then proceeded to initiation of chemotherapy.

For the patients in the first trimester, administration of cytotoxic chemotherapeutic agents during pregnancy has been found to be associated with unfavorable fetal outcomes, including spontaneous abortion, intrauterine fetal death, and major malformations in 10–20% of patients [22,33]. The critical period for teratogenicity of chemotherapy is between week 3 and 10 of gestation, since this period correlates with active organogenesis and foetal development [34]. High teratogenicity of cytotoxic agents during pregnancy had been shown in some animal experiments [8]. Standard protocols to treat AML include anti-metabolite cytarabine and an anthracycline [32]. Daunorubicin is the anthracycline of choice, because it may induce less foetal toxicity than idarubicin, a derivative of daunorubicin. Idarubicin is more lipophilic, longer half-life, and with increased placental transfer [32]. However, both cytarabine and daunorubicin are well recognized to cause foetal abnormalities, including limb deformities, ventral septal defect, and cardiomyopathy [12]. If AML presents during the first trimester, a successful pregnancy is unlikely [7], therefore it is strongly recommended to terminate pregnancy rather than allowing spontaneous abortion during a potential thrombocytopenic or neutropenic phase [3]. When AML occurs in the second and third trimesters, chemotherapy with daunorubicin and cytarabine can be successfully administered and should start without delay [3], with extensive and continuous monitoring of foetus vital signs, cardiac function and congenital abnormalities (especially limb development). For ALL patients, the standard regimen of induction therapy consists of 4-drug or 5-drug for 4 weeks, which makes the management further complicated. Therefore, termination of pregnancy should be considered before conventional therapy for ALL patients diagnosed in first trimester and early second trimester (before 20 weeks’ gestation) [12,35]. Additionally, as an important part of ALL therapy, methotrexate, is highly teratogenic and contraindicated in the first and second trimester [22,36]. Although intensive chemotherapy is crucial for the prognosis of acute leukaemia, there is evidence that proper modification of the chemotherapy regimen during pregnancy benefits the patient without affecting the health of the fetus [32]. In our study, four AML patients diagnosed before 20 weeks in the second trimester all opted for therapeutic abortion, due to worry about the harmful effect of cytotoxic agents on the fetus.

APL, the M3 subtype of AML, is always associated with coagulopathy. ATRA, a pivotal drug for APL treatment, is highly teratogenic and administered in the first trimester led to about 14% malformation [37]. Use of ATRA during the first trimester has also been associated with miscarriage in 40% of patients [38,39]. Consequently, the current recommendation for the early stage of pregnancy is to reject the use of ATRA [12]. While treatment with ATRA alone during the second and third trimester until complete remission was proposed for low-risk patients, delaying the administration of chemotherapy (or arsenic trioxide) until delivery [23,40,41]. In our study, patient No.2 diagnosed in the first trimester received ATRA and DNR therapy followed by planned therapeutic abortion. Patient No.14 diagnosed with high-risk APL (WBC count >10 × 10^9^/L) at 34 weeks started induction therapy (ATRA + DNR) first and delivered a full-term baby at 38 weeks. While patient No.16 diagnosed with low-risk APL at 35 weeks opted to receive ATRA alone and had a delivery of a full-term healthy baby, then initiated IDA cytoreductive chemotherapy because of a marked increase in WBC counts. All of these 3 patients achieved CR. Arsenic trioxide (ATO) is another effective drug for treatment of APL, and its application has made considerable improvement in APL therapy over the past two decades [42]. However, ATO is highly embryo toxic and is contraindicated at any stage of pregnancy due to increased risk of fetal malformations, intrauterine growth restriction, stillbirth and spontaneous abortions [41,43,44]. While the combination of ATRA and ATO is recommended for patients after termination or delivery, it’s noted that breastfeeding is contraindicated in this case.

Unfortunately, four patients died before chemotherapy. This result may suggest that, symptoms caused by acute leukemia, such as fatigue, pale and shortness of breath, or alterations of peripheral blood counts, might be misinterpreted as pregnancy-related symptoms [23], resulting in delayed diagnosis. Early diagnosis and treatment may improve the outcome of these patients.

Nine healthy babies were delivered, four of them exposed to chemotherapeutic agents. It has been reported that fetal exposure to chemotherapy is associated with an increased risk of low birth weight [45]. In the present study, all of the nine were low-birth-weight newborns, including 5 full-term infants. The main reason may be maternal anemia and/or nutritional deficiencies, caused by leukemia and chemotherapy-induced anorexia [32].

**Figure 1.**
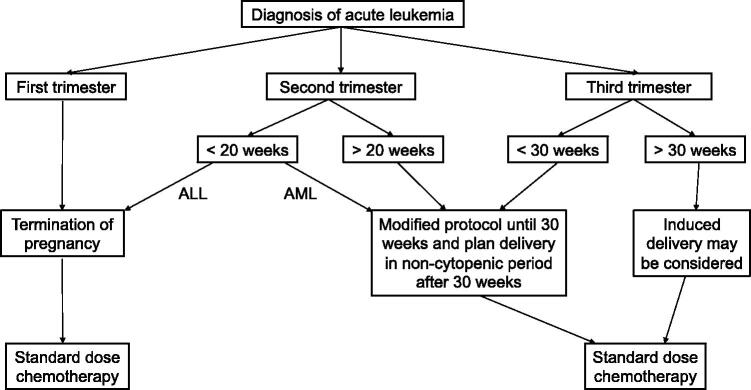
Proposed management of acute leukemia during pregnancy.

In general, for patients with acute leukemia diagnosed during pregnancy, initiation of chemotherapy as soon as possible may increase the CR rate. While for patients who diagnosed in the first trimester, elective termination followed by standard-dose chemotherapy is a good choice to ensure better maternal outcomes. For patients diagnosed in the advanced stage of pregnancy (>30 weeks’ gestation), induced delivery before starting chemotherapy to reduce fetal chemotherapy exposure may be reasonable for better maternal and fetal outcomes. The proposed treatment strategy is summarized in Figure 1.

## Limitation

The diagnosis and management of acute leukaemia in pregnancy remain a substantial challenge. Due to the characteristics of pregnancy, we are unable to prospectively study the most appropriate management for patients diagnosed with AL during pregnancy.

## Future prospects

Given the rarity of pregnancy with acute leukemia, the experience of treating this dilemma is limited. Therefore, it is urgent to establish a national or multicenter database to collect the information of patients with pregnancy complicated with acute leukemia. The existence of such a database will provide a better basis for clinical studies and epidemiological follow-up, and also provide a better reference for the development of future guidelines and standardized treatment.

## Supplementary Material

Supplemental Material
