# Supplementary material for: Acute leukemia in pregnancy: a single institutional experience with 21 cases at 10 years and a review of the literature
Source: Ann Med. 2021 Apr 6;53(1):567–75. doi: 10.1080/07853890.2021.1908586 (PMC8032338; doi:10.1080/07853890.2021.1908586)
Supplement: Supplemental Material [file IANN_A_1908586_SM6236.docx]

Table S1. Laboratory findings of 21 patients with acute leukemia diagnosed during pregnancy

| Patient No. | Age at diagnosis (years) | AL type | Peripheral blood counts at diagnosis | | | Blasts in BM（%） | Cytogenetics | Molecular markers |
| --- | --- | --- | --- | --- | --- | --- | --- | --- |
|  |  |  | WBC (×10^9^/L) | HB (g/L) | PLT (×10^9^/L) |  |  |  |
| 1 | 33 | AML-M5 | 57.26 | 53 | 23 | 64 | 46, XX, -7q[15]/46, XX[5] |  |
| 2 | 25 | AML-M3 | 0.97 | 105 | 55 | 19 | 46, XX, t(15;17)[7]/46, XX[13] | PML-RARa(+) |
| 3 | 30 | AML-M5 | 37.8 | 71 | 63 | 63 | 46, XX[20] |  |
| 4 | 19 | ALL | 27.3 | 87 | 65 | 76 | 46, XX[20] | BCR-ABL(-) |
| 5 | 33 | ALL | 34.3 | 82 | 10 | 29 | 47, XX, +22[6]/46, XX[14] | BCR-ABL(-) |
| 6 | 34 | AML-M2 | 49.4 | 61 | 14 | 84 | 46, XX, t(8;21)(q22;q22)[20] | AML1-ETO(+) |
| 7 | 28 | AML-M4 | 36.2 | 93 | 67 | 65 | 46, XX[13] |  |
| 8 | 39 | AML-M4 | 8.07 | 51 | 36 | 39 | 46, XX, inv(16)(p13;q22)[10] | CBFb-MYH11(+), NPM1 mut(+) |
| 9 | 26 | AML-M1 | 144 | 54 | 67 | 88 | 46, XX[7] |  |
| 10 | 23 | AML-M4 | 118.25 | 84 | 12 | 20.5 | 46, XX[20] |  |
| 11 | 40 | AML-M4 | 45 | 42 | 74 | 29 | 45-46, XX, t(3;4) (p21;p16),t(7;11)(q22;q18), del(7)(q22), del(p11.2),add(9)(q34), Ins(12:?)(q13:?),del(22)(q11)[cp20] |  |
| 12 | 24 | AML-M4 | 3.94 | 118 | 64 | 77 | 46, XX[3] | FLT3-ITD(+), NPM1 mut (+) |
| 13 | 28 | AML-M5 | 2.9 | 105 | 60 | 68 | 46, XX, add(15)(q26), del(20)(q11)[cp18]/46,XX[2] |  |
| 14 | 27 | AML-M3 | 23.58 | 90 | 92 | 95 | 46, XX, t(15;17)(q22;q21)[10] | PML-RARa(+) |
| 15 | 41 | ALL | 142.98 | 54 | 12 | 88 | Failure | BCR-ABL(-) |
| 16 | 24 | AML-M3 | 0.79 | 54 | 12 | 68.5 | 46, XX, t(15;17)[20] | PML-RARa(+) |
| 17 | 26 | AML-M2 | 13.59 | 103 | 146 | 68 | 46, XX[20] | FLT3-ITD(+), CEBPA mut(+) |
| 18 | 30 | AML-M3 | 8.04 | 63 | 19 | 58 | 46, XX, t(15;17)[6]/46, XX[14] | PML-RARa(+) |
| 19 | 29 | AML-M2 | 5.24 | 72 | 16 | 33 | 46, XX[20] |  |
| 20 | 27 | AML-M2 | 2.26 | 95 | 240 | 24.5 | 46, XX, t(8;21)(q22;q22)[6]/46, XX[14] | AML1-ETO(+), IDH-R140 (+) |
| 21 | 26 | AML-M4 | 43.12 | 58 | 20 | 21 | 46, XX[15] | HOX11(+) |

AL: acute leukemia; WBC: white blood cell; HB: hemoglobin; PLT: platelet; BM: bone marrow; AML: acute myeloid leukemia; ALL: acute lymphoblastic leukemia.
